# Draft genomes for one *Microcystis*-resistant and one *Microcystis*-sensitive strain of the water flea, *Daphnia pulicaria*

**DOI:** 10.1093/g3journal/jkab266

**Published:** 2021-08-16

**Authors:** Amanda D Clark, Bailey K Howell, Alan E Wilson, Tonia S Schwartz

**Affiliations:** 1 Department of Biological Sciences, Auburn University, Auburn, AL 36849, USA; 2 Bioinformatics REU Program, Department of Biological Sciences, Auburn University, Auburn, AL 36849, USA; 3 Department of Biological Sciences, Virginia Polytechnic Institute and State University, Blacksburg, VA 24061, USA; 4 Fisheries, Aquaculture and Aquatic Sciences, Auburn University, Auburn, AL 36849, USA

**Keywords:** toxic algae, freshwater ecology, genomics, cyanobacteria, toxin resistance

## Abstract

*Daphnia* species are well-suited for studying local adaptation and evolutionary responses to stress(ors) including those caused by algal blooms. Algal blooms, characterized by an overgrowth (bloom) of cyanobacteria, are detrimental to the health of aquatic and terrestrial members of freshwater ecosystems. Some strains of *Daphnia pulicaria* have demonstrated resistance to toxic algae and the ability to mitigate toxic algal blooms. Understanding the genetic mechanism associated with this toxin resistance requires adequate genomic resources. Using whole-genome sequence data mapped to the *Daphnia pulex* reference genome (PA42), we present reference-guided draft assemblies from one tolerant and one sensitive strain of *D. pulicaria*, Wintergreen-6 (WI-6), and Bassett-411 (BA-411), respectively. Assessment of the draft assemblies reveal low contamination levels, and high levels (95%) of genic content. Reference scaffolds had coverage breadths of 98.9–99.4%, and average depths of 33X and 29X for BA-411 and WI-6, respectively. Within, we discuss caveats and suggestions for improving these draft assemblies. These genomic resources are presented with a goal of contributing to the resources necessary to understand the genetic mechanisms and associations of toxic prey resistance observed in this species.

## Introduction

Over the past two decades, functional ecology research has focused on constructing a theoretical framework for eco-evolutionary dynamics, the bidirectional feedback between ecological and evolutionary processes of populations, communities, and ecosystems (Pelletier *et al.* 2009). An integral portion of this framework requires connecting genetic variation in species and the concomitant effects on ecological interactions across hierarchal levels (Brunner *et al.* 2019). Ideal species for studying this interplay of evolution and ecology possess high connectivity within their ecological communities (see Figure 2 in Miner *et al.* 2012), experimental flexibility, and tractability (*i.e.*, responds to a multitude of diverse stressors; ease of controlled maintenance and manipulation), and suitable genomic resources (Miner *et al.* 2012). *Daphnia*, commonly known as water fleas, satisfy these criteria. *Daphnia* is well-studied, widely employed models in ecology, evolution, and ecotoxicology (Shaw *et al.* 2007; Eads *et al.* 2008; Sarnelle *et al.* 2010; Miner *et al.* 2012; Asselman *et al.* 2018; Becker *et al.* 2018; Nelson *et al.* 2018), and have been utilized in Nobel prize-worthy discoveries (Nobel Media AB 2021).

*Daphnia* species, with the appealing traits of short generation times and cyclic parthenogenesis, are well-suited for studying local adaptation and evolutionary responses to stress(ors) including those caused by global warming and anthropogenic eutrophication (Hairston *et al.* 2001; Ebert 2005; Asselman *et al.* 2014). *Daphnia pulicaria*, a lake-dwelling herbivorous zooplankton in the genus, demonstrate evidence of local adaptation to cyanobacteria in eutrophic lakes and significant genetic structure amongst populations (Sarnelle and Wilson 2005; Chislock *et al.* 2019a). *Microcystis aeruginosa* is a highly toxic species of cyanobacteria, abundant in harmful algal blooms, that may produce toxic metabolites, including a suite of hepatotoxins called microcystins (Paerl *et al.* 2001). Many methods for controlling these blooms have been proposed due to their adverse effects on human health, the economy, and ecological communities. One promising avenue for mediation is biomanipulation or manipulating trophic levels to control cyanobacterial overgrowth by introducing *D. pulicaria* exhibiting resistance to toxic cyanobacteria (Wilson and Chislock 2013; Chislock *et al.* 2019b). These findings have contributed to a strong, growing interest in using *D. pulicaria* to understand the mechanistic link between genetic trait variation and ecological community dynamics, which could aid in informing mitigation tactics for harmful algal blooms. However, such efforts require increasing the available genomic resources.

Currently, there are full genomes assemblies for four *Daphnia* species*: D. pulex*, TCO (Colbourne *et al.* 2011) and PA42 (Ye *et al.* 2017); *D. magna*, KIT (Lee *et al.* 2019) and XINB3 (Gilbert, D.G, unpublished [PRJNA298946]); *D. carinata*, WSL (Jia *et al.* 2020); and a *D. galeata* assembly [PRJEB42807] (Nickel *et al.* 2021). Published whole genome amplifications of single and pooled *D. pulicaria* adult and ephippia have been mapped to the *D. pulex*TCO genome assembly, but the genomic resources presented here are the first genome assemblies for *D. pulicaria* assembled using the new and improved, *D. pulex* PA42 genome assembly (Lack *et al.* 2018). Here, we present two reference-guided assemblies from two strains of *D. pulicaria*, one *Microcystis*-resistant strain, Wintergreen-6 (WI-6), and one *Microcystis*-sensitive strain, Bassett-411 (BA-411).

## Materials and methods

### Samples

Two strains of *D. pulicaria*, WI-6, and BA-411, were initiated from a single individual isolated from small glacial lakes in southern Michigan during autumn 2004 and spring 2009, respectively (Chislock *et al.* 2019a, 2019b). Tolerant phenotypes were established by exposing neonates to toxic cyanobacterial diets where strains from highly eutrophic lakes, like WI-6, demonstrated reduced negative impacts on growth rates (Sarnelle and Wilson 2005). These strains have been maintained in clonal cultures in the freshwater ecology laboratory of Dr. Alan Wilson (AU) since isolation and were received from the Wilson Lab in December of 2018 for genome sequencing. For each strain, approximately 10–15 individuals were cultured in autoclaved 50 ml flasks loosely capped with foam stoppers and transferred to fresh food and water on a biweekly basis. Clonal populations for each strain were cultured at (23°C) room temperature in (autoclaved) water from a nearby, oligotrophic reservoir (Lake Martin, AL, USA), and fed a nutritious alga, *Ankistrodesmus falcatus*, *ad libitum*. As populations reproduced, offspring were quantified and separated into new flasks on a weekly basis. Offspring were allowed to mature before being transferred into diethylpyrocarbonate (DEPC)-treated water [VWR, USA] with no food for 2 days in order to clear their guts. Post-starvation, 20 adults were pooled into 1.5 ml tubes in 1 ml of a 1.5x DNA/RNA Shield [Zymogen, USA] and stored at 4°C. For each strain, we used three tubes of 20 adult *Daphnia* for DNA extractions for genome sequencing.

### DNA extraction

DNA was extracted within 24–48 h of DNA/RNA Shield storage using the QIAamp UCP DNA Micro kit [QIAGEN, Germany] per manual instructions, with some modifications. Briefly, DNA/RNA Shield was removed, 10 µl of kit proteinase k (half the recommended amount) and two 2.0 mm silicate beads were added before samples were homogenized on a TissueLyser II [QIAGEN] for 1 min at a frequency of 30 cycles(s^−1^). The remaining steps of the manufacture’s protocol were followed with DNA being eluted from the filter in a 20 µl volume. Independent DNA extractions from were performed over 3 weeks during March 2019 were frozen at −20°C. For each strain, the DNA from three samples were pooled and concentrated in preparation for genome sequencing, thus the genomic sequence represents approximately 60 individuals, that are presumed to be clonal. DNA was quantification using the Qubit dsDNA High Sensitivity Assay kit [Thermo Fisher, USA].

### Validation of strain via genotyping PCR

To validate that DNA samples were of the correct and single strain of origin, samples were PCR amplified for *DP496*, a microsatellite locus previously identified in Colbourne *et al.* (2004) and demonstrated to have discriminating allelic patterns between these two *D. pulicaria* strains (Wilson and Hay 2007; Chislock *et al.* 2019a). Primer sequences were obtained from the *Daphnia* Genomics Consortium, wfleabase (http://wfleabase.org/genomics/microsatellite/) (Colbourne *et al.* 2004, 2005). PCR reactions were carried out in 10 µl volumes using 5 µl of 2X GoTaq Green PCR Master Mix [Promega, USA], 0.3 µl of 10 µM forward and reverse primers (0.3 µM final concentration), 3.65 µl of water, and 0.75 µl of DNA (21 ng). The thermocycler program for the PCR began with a 2 min denaturation cycle at 95°C, followed by 35 cycles of 20 s at 95°C, 20 s at 50°C, and 20 s at 72°C, and a final extension cycle for 10 min at 72°C. DNA from a single individual of each strain was used as positive controls, and water instead of DNA was used as a no template control. Five microliters of PCR products were visualized on a 3% agarose gel made with 0.5X TAE and 1 µl of GelGreen Nucleic Acid Stain [Biotium, USA] to confirm that the allelic patterns for the genome samples were consistent with the positive controls for the target strains.

### Sequencing

For each strain, approximately 0.8 µg (WI-6) and 1 µg (BA-411) of DNA were shipped to Novogene [China] for sequencing. Novogene performed library preparation using the Illumina TruSeqLibrary Construction Kit and sequencing on an Illumina Novoseq 6000, producing 8 Gbs (54.8 and 56.1 million reads for BA-411 and WI-6, respectively) of 150 bp PE reads.

### Reference-guided assembly

For each strain, we conducted reference-based assembly using the *D. pulex* PA42 genome assembly. *D. pulex* was determined to be a suitable, high-quality reference, as it is closely related to *D. pulicaria* and, interestingly, the two commonly hybridize in ecological communities (Marková *et al.* 2013; Kake‐Guena et al. 2015). Furthermore, the PA42 genome was produced from starved *Daphnia* treated with antibiotics to reduce diet and endosymbiotic contaminants, and post-assembly, scaffolds were filtered for bacterial contamination (Ye *et al.* 2017). Our assembly pipeline, described below, was run on Auburn University’s High-Performance Cluster, Hopper for 2 days using 20 cores and 100GB of memory.

For each strain, we used the following pipeline. Quality assessment of raw data files was performed using *FASTQC* v0.11.5 (Andrews 2010). The assessment reported no adapter contamination and no regions where sequence quality dropped below Q-score of 25, therefore trimming was not applied to reduce unnecessary loss of data. *D. pulicaria* reads were mapped to PA42 using Burrows-Wheeler Aligner (*BWA)* v0.7.15 (Li and Durbin 2009). Genome Analysis Tool Kit (*GATK)* v3.6 was used for local realignment, insertion/deletion (INDEL), and single nucleotide polymorphism identification, and separation and filtration of identified variants using GATK recommended hard-filtering parameters (McKenna *et al.* 2010; der Auwera *et al.* 2013). SNPs were inserted into the original reference, creating a consensus sequence, using *BCFTools* “*consensus*” (Li 2011). *BEDTools* “*genomcov*” was used to create a BED file of regions lacking reference read coverage and “*maskfasta*” was used to mask the zero-coverage and INDEL regions in the consensus sequence with “N’s” (Quinlan and Hall 2010). This produced a reference-guided, draft genome assembly for each strain.

### Assembly metrics and assessments

Although we starved the *Daphnia* before sequencing, it is likely there was still remnant algal cells and bacterial contaminates in our sequencing data. To identify these and any other contaminates, *BlobTools* (v 1.0) workflow A was used to quantify and visualize represented taxa, therefore identifying contamination from other phyla in the raw reads included in the draft assemblies (Laetsch and Blaxter 2017). *FastQ Screen* was also used as a screening method for contaminants by mapping a subset of raw reads to a search library with *bowtie2* (Langmead and Salzberg 2012; Wingett and Andrews 2018). The search library genomes included with the program were used and genomes for PA42, the *D. pulex* mitochondria (PRJNA11866), and the green algae *Monoraphidium neglectum* (PRJNA293989), a close relative of the food source for *Daphnia*, were added (Crease 1999; Bogen *et al.* 2013). Assembly completeness was estimated with Benchmarking Universal Single-Copy Orthologs (*BUSCO*) v4.0.6 analysis using both the eukaryote_odb10 and arthropoda_odb10 databases (Simão *et al.* 2015; Waterhouse *et al.* 2018). *BEDTools* “*coverage*” was also used to determine depth of coverage at genes annotated in PA42*. Sourmash* v4 uses a MinHash derived algorithm to estimate similarity of genomic sequences and was used here to make pairwise comparisons between draft and reference assemblies (Brown and Irber 2016). *Sourmash* was used to create DNA sketches, or hash sketches, from both the assemblies and the merged raw reads. These reduced sequence data representations can be rapidly compared for overlapping k-mer sized read content (overlapping k-mer space) using a Jaccard similarity coefficient, however, it does not give information about genomic contiguity or structure. Based on recommendations in *sourmash* documentation, signatures were computed for k-mer sizes of 21, 31, and 51 bp, to minimize false positives and maximize matches. The distance measure output from this method is highly correlated with the frequently used genetic distance measurement, average nucleotide identity (ANI) (Ondov *et al.* 2016).

## Results and discussion

### Assemblies

PA42 is a quality *D. pulex* genome consisting of approximately 156 megabase pairs (8.6% gaps) organized into 1822 scaffolds. The BA-411 sequencing library produced 54.8 million reads with 24.9% duplication, and the WI-6 library produced 56.1 million reads with 21.7% duplication. Using BWA, BA-411 and WI-6 reads were mapped to PA42 resulting in approximately 86 and 75% successfully mapped reads, respectively. Of the 1822 scaffolds making up the PA42 assembly, 21 (1.15%) and 12 (0.66%) reference scaffolds had no sequence coverage for BA-411 and WI-6, with average coverage depths of ∼33X and ∼29X, respectively for the rest of the assembly. Assembly metrics are compared in Table 1. Although *D. pulex* and *D. pulicaria* are closely related, the assemblies presented herein are reference-guided and regions of the genomes that are not truly syntenic between the species will be incorrect in these BA-411 and WI-6 draft assemblies.

**Table 1:** Mapping and base calling statistics for *Daphnia pulicaria sequencing libraries and* reference-guided assemblies

Location	Library	GDNA CONC. [ng/µl]	Reads generated	Reads mapped	% Reads mapped	Mean depth	Called sites	N’s	PA42 Mb covered	SRA accession
Bassett Lake	USD16091408	28	54,849,146	47,627,534	86.83	33.55	2,923,235	28,692,854	140	SRR14023941
Wintergreen Lake	USD16091409	21.6	56,113,536	42,293,065	75.37	29.46	3,070,431	27,250,307	141	SRR14023940

**Figure 1:**
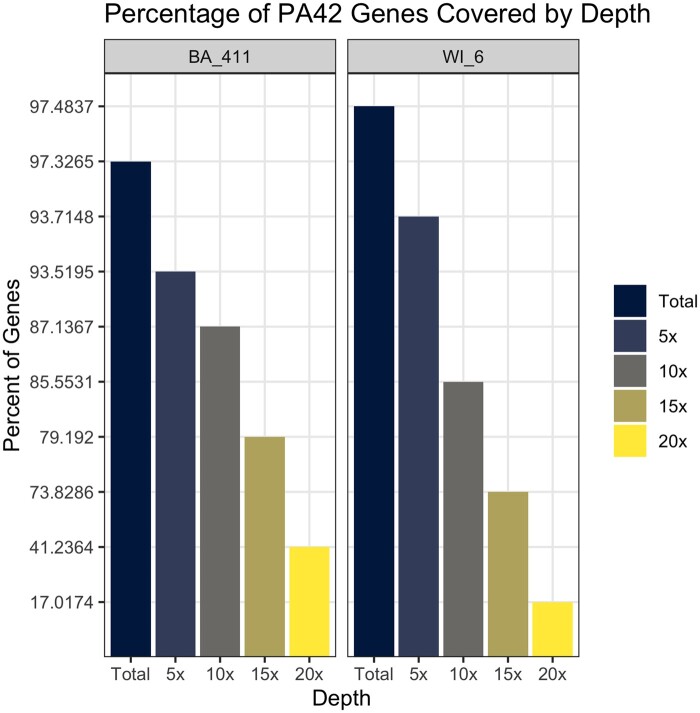
Percent of PA42 genes for different average depths of coverage. The left and right panels represent BA-411 and WI-6, respectively. “Total” is the number of genes with atleast 1x average depth. The percentage of genes covered at average depths of 5, 10, 15, and 20x are included.

Approximately 97% of genes annotated in PA42 had coverage from mapped BA-411 and WI-6 reads. The percentage of PA42 genes with atleast 1X coverage is represented by the “Total” category in Figure 1. The percentage of PA42 genes with 5, 10, 15, and 20X average depth are also found in this figure. Interestingly, BA and WI-6 have a similar number of genes with coverage, but WI-6 has consistently fewer genes covered at average depths of 10x or higher.

### Assessments

**Figure 2:**
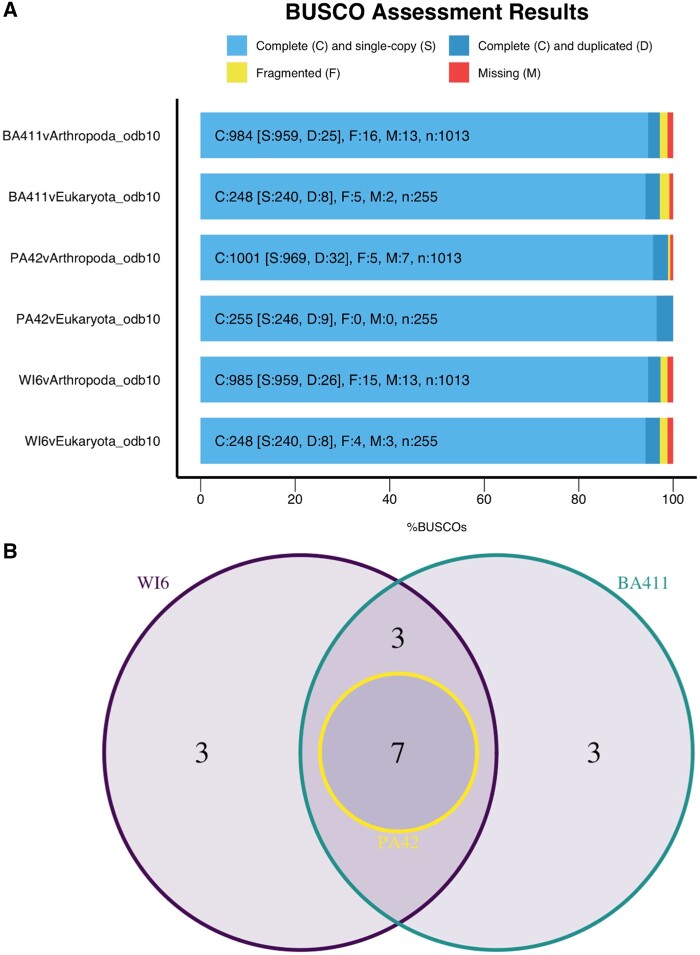
BUSCO analysis for *D. pulicaria* genome assemblies indicates high levels of gene content in draft assemblies. (A) BUSCO analysis for draft assemblies, BA-411 and WI-6, and the reference genome, PA42, against the eukaryote and arthropod databases. Colors indicate status of ortholog in the assembly. (B) Venn diagram of missing arthropod BUSCOs for three *Daphnia* assemblies. Seven of the 13 missing BUSCOs in *D. pulicaria* assemblies were not present in the PA42 reference genome used for assembly.

To further assess the completeness of BA-411 and WI-6 assemblies, a *BUSCO* analysis was run using both the arthropod and eukaryote databases. Over 95% of the universal single-copy orthologs searched from both databases were found to be complete in both draft assemblies, with a minor difference in fragmented orthologs (0.1%; Figure 2A). The Venn diagram of missing BUSCOs in Figure 2B indicates that there are seven missing across all assemblies, corresponding to what is missing in the reference, and six BUSCOs that are missing in *D. pulicaria* only, with three species-specific orthologs missing in both strains and three strain-specific orthologs missing from each strain. These data indicate high contiguity in many genic regions for these draft assemblies.

**Figure 3:**
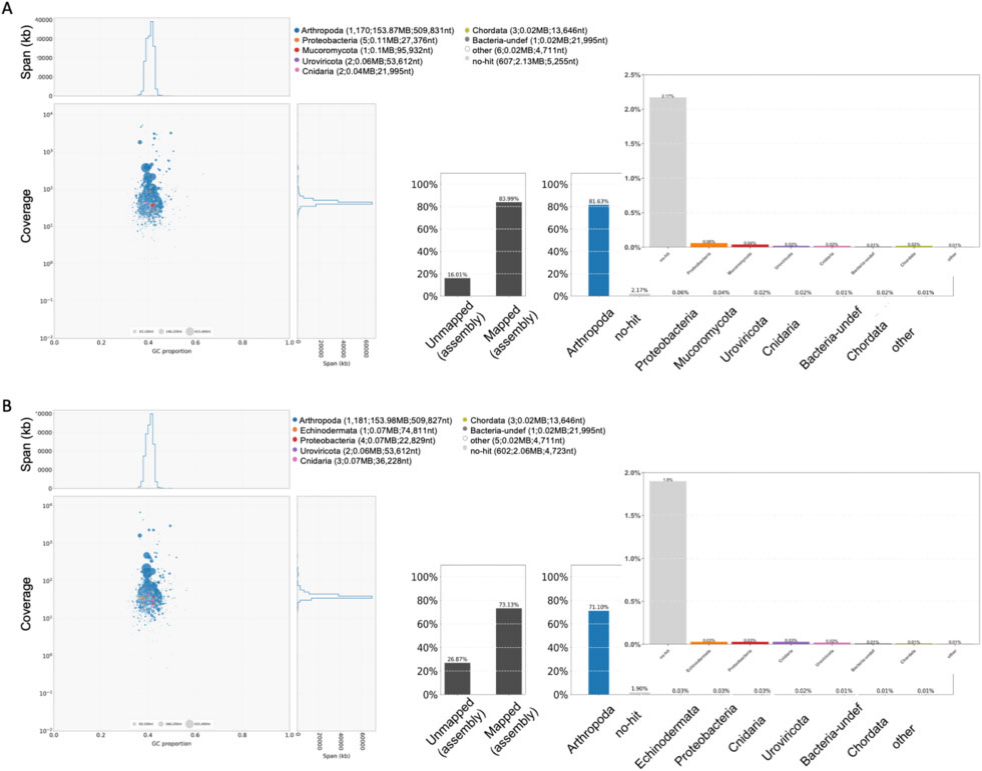
BlobPlots indicate low levels of contaminant Phyla in BA411 and WI6 draft reference-guided assemblies. Coverage by GC content scatterplots (BlobPlot) accompanied by read coverage plots for (A) BA-411 and (B) WI-6 draft assemblies. BlobPlots: The circles are the sequences, with sequence length proportional to circle diameter. The legend indicates each phyla represented with count, total span, and N50 for each taxonomic rank in parentheses. Only a small number of sequences used in BLASTx analysis against the NCBI nonredundant protein database hit to phyla (19) other than the target Arthropoda. BarPlots: The dark gray bars represent the proportion of unmapped and mapped reads from libraries. Color bars represent the mapped proportion by taxonomic rank (phyla); an inset is included for viewing taxa present at low proportions with the light gray bars representing reads that had no hits in the database.

Assemblies were assessed for contamination with *BlobTools*. We had an expectation of bacterial and algal contamination in the read data considering the microenvironment and diet of *Daphnia*, but because we used a reference sequence where great measures were taken to remove contaminants, we expected that a vast majority of contaminants would be filtered out during mapping. Based on the blob plots (Figure 3), both drafts genome assemblies had low levels of contaminant sequences, with 0.22% of BA-411 and 0.13% of WI-6 mapped reads hitting to phyla outside of Arthropoda. Supplementary data include *BlobTools* output to further explore or remove contaminant regions.

*FastQ Screen* results (Supplementary Figure S1) corroborate the *BlobTools* analysis with a majority of the raw read subset mapping to the PA42 library. A portion of the read subsets (19–30%) mapped at low, nonspecific levels or did not map at all to the other species and sequences included in the search library. This suggests that the appropriate search genome was not included, and it is likely that these reads may be unique to *D. pulicaria* or that a completely unexpected contaminate is present.

To gain a preliminary perspective on genetic distance between the two draft assemblies and the PA42 reference, we used *sourmash*. Distance estimates range from zero, being completely divergent to one, being completely identical. The assemblies from the *D. pulicaria* strains BA-411 and WI-6 had a computed distance of 0.90. This result is intuitive, as these are two strains of the same *Daphnia* species. BA-411 and WI-6 had very similar distance estimates for PA42 comparisons, with estimates of 0.73 for BA-411 and 0.74 for WI-6 (Supplementary Figure S2). These results corroborate the slight increase in gene content and PA42 scaffold coverage obtained from *BUSCO* analysis and mapping statistics for WI-6.

## Conclusions

*Daphnia* species have long been studied in the context of ecology, evolution, and applied research. Here, we present draft genome assemblies for two strains of *Daphnia* that vary in their tolerance to cyanobacteria. Algal blooms, characterized by an overgrowth (bloom) of cyanobacteria, are detrimental to the health of aquatic and terrestrial members of freshwater ecosystems. Population expansion of cyanobacteria is caused by eutrophication, or the overloading of nutrients (*e.g.*, phosphorus and nitrogen) in lakes, ponds, and rivers, and is accelerated by increasing temperatures (Carpenter 2005; Schmale *et al.* 2019). From an economic perspective, algal blooms decrease water quality due to decreases in available oxygen and increases in toxic metabolites produced by cyanobacteria that result in product losses in fisheries, and toxification of water sources used by wild, domestic, and human populations for consumption and aquatic recreation (Anderson *et al.* 2002; Schmale *et al.* 2019). *M.* *aeruginosa* is a highly toxic, cosmopolitan species of cyanobacteria that may produce metabolites called microcystins, compounds demonstrated to have significant hepatotoxic and tumorigenic effects (Paerl *et al.* 2001). Keeping levels of these damaging algal blooms in check is a particularly important and active branch of ecological research. Methods proposed for managing cyanobacteria include reducing the introduction of extraneous nutrients often from human runoff, the introduction of herbicide, and biomanipulation, or the manipulation of trophic levels to control cyanobacteria populations. Introducing toxin-tolerant *D.* *pulicaria* has been shown to repeatedly lead to significant reductions of total algal biomass, including cyanobacteria, in limnocorral experiments (Wilson and Chislock 2013; Chislock *et al.* 2019a, 2019b).

In addition to understanding *D. pulicaria’s* top-down regulation of algal biomass through mesocosm experiments, we are building resources to understand the genetic mechanisms and associations of toxic prey resistance observed in this species with these draft assemblies. Genomic resources are key components to deepening our understanding of the contributions of genetic background on strain-specific responses to toxic algal blooms and other environmental stressors. These resources can be used for understanding the transcriptomic responses to toxins (Asselman *et al.* 2012; Orsini *et al.* 2016; Giraudo *et al.* 2017), identifying sequence variants under positive selection across the genome (Bourgeois *et al.* 2017; Schwarzenberger *et al.* 2020), and comparative analysis across other *Daphnia* species (Ravindran *et al.* 2019). In this way, these genomic resources provide a promising avenue for future research as the effects of urbanization and global climate change continue to exacerbate the severity of these toxic algal blooms over time (Carpenter 2005; Schmale *et al.* 2019).

These are reference-based *D. pulicaria* draft genome assemblies. In this study, 14–25% of the reads did not map to *D. pulex* PA42 genome assembly in our mapping. Similar to the read mapping percentages reported here (75–86%), Lack et al. (2018) produced sequencing libraries for pooled and individual *D. pulicaria* adults and ephippia and reported an average of ∼72% mapping success to the TCO reference genome across 11 libraries (Lack *et al.* 2018). This indicates room for improvement in our assemblies. The data presented here are short-read sequences (150 bp paired-end). Future analyses should include long-read sequence data appropriate for *de novo* assembly that could recover the unmapped regions, improve scaffolds presented here, identify novel *D. pulicaria* scaffolds and chromosomal rearrangements to resolve conflicts in genetic structure between *D. pulex* and *D. pulicaria* genomes. Even with the aforementioned caveats of the two *D. pulicaria* genome assemblies we present, these assemblies contain very low levels of contamination, and high levels of genic content with more than 95% of complete universal arthropod and eukaryote orthologs found in these assemblies. This work contributes quality reference-guided assemblies for two strains, one tolerant and one sensitive, of *D. pulicaria* that can be useful resources in linking candidate genes involved in ecologically relevant trait divergence, such as the evolution of dietary tolerance to toxic cyanobacteria, that impact freshwater communities and ecosystems.

## Data availability

Supplementary files can be found on GSA figshare (https://doi.org/10.25387/g3.14978991). Supplementary File S1 contains Figure S1 of the *FastQ Screen* analysis and Figure S2 of the *Sourmash* distance estimates between the assemblies. Supplementary File S2 is a tarball containing the reference-guided assemblies for BA_411 and WI_6. Assembly files with “clean” appended to the name have been filtered for scaffolds without reference coverage. Supplementary File S3 is a tarball containing *Blobtools* output. Supplementary File S4 is a tarball containing BUSCO outputs. All sequence data are available under the NCBI BioProject Accession PRJNA702463. Code used to perform the data analyses for this work can be found on GitHub (https://doi.org/10.5281/zenodo.4635402).
